# Optical tuner for sustainable buildings

**DOI:** 10.1038/s44172-023-00072-5

**Published:** 2023-05-05

**Authors:** Mengying Su

**Affiliations:** Communications Engineering, https://www.nature.com/commseng

**Keywords:** Engineering

## Abstract

A recent publication in *Proceedings of the National Academy of Sciences* describes a fluid-based building interface to reduce energy use for heating, cooling and lighting by selectively tuning light absorption and dispersion. Models showed that this system could reduce the annual energy consumption by up to 43% compared to existing technologies.

**Figure:**
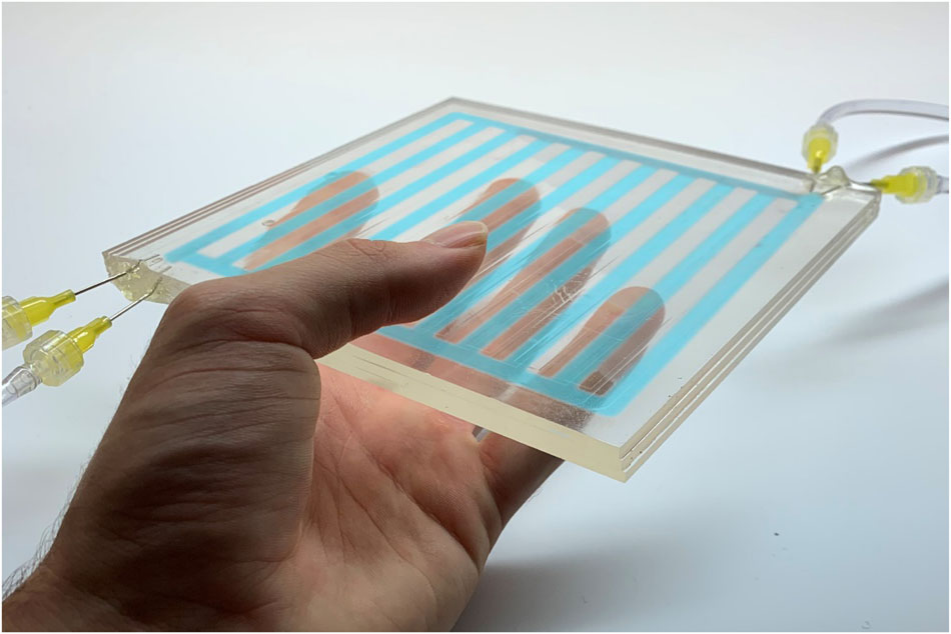
Raphael Kay and Adrian So

Buildings represent a critical component of the carbon footprint of cities. According to IPCC’s (Intergovernmental Panel on Climate Change) fifth assessment report (https://www.ipcc.ch/site/assets/uploads/2018/02/ipcc_wg3_ar5_chapter9.pdf), in 2010, buildings accounted for 32% of total global energy use and 19% of total energy-related greenhouse gas emissions. These numbers are continuously increasing with urbanization, and might be doubled or tripled by mid-century. Heating and cooling of space and water in buildings is the largest energy end-use, contributing 46% of the total global heat use (https://www.iea.org/reports/renewables-2019/heat). More cost-effective techniques are needed to improve energy efficiency.

Current solar thermal regulation techniques are based on tuning three optical properties of sunlight- intensity, wavelength and dispersion. However, these technologies are only able to partially control each of these three optical properties. For example, certain reflective coatings can often only modulate wavelength, while electrochromic devices can usually only regulate intensity. A system capable of independent as well simultaneous control over multiple relevant optical properties is needed.

In nature, certain biological species have evolved skin with multiple layers which can independently tune various solar adaptations. Raphael Kay, Ben Hatton and collaborators at the University of Toronto adopted this bio-inspired optical control strategy in an optofluidics platform to achieve independent combinatorial control of the transmitted light intensity, absorption and distribution within buildings.

Similar to the organic structures with different absorption spectra and scattering responses in biological skin, liquid solutions with various colors and particles could achieve a similarly versatile range of functions. The researchers fabricated centimetre-scale bilayer and trilayer devices using transparent millimetre-thick polymethylmethacrylate (PMMA) sheets. Each layer was designed with stacked parallel-channels and fluidic reservoirs at the end of each channel. Dyed solutions were pumped in and out for switchable and reversible optical control. For example, two parallel layers with blue and yellow dye solutions transmitted light with an effective green color. Thus, the confined flows within different layers could achieve spectrally selective absorption of visible and near-infrared-selective (NIR) light as well as direct light scattering by filling the channels with different solutions.

Raphael Kay and colleagues experimentally demonstrated the feasibility of this fluid-based device for NIR-selective absorption and light intensity reduction using dyes in different colors and carbon black pigments. Numerical simulations showed that the fluid multilayers could reduce the annual electric lighting energy consumption by 21% and 24% over electrochromic and roller shade systems respectively. Energy consumption for heating during the winter was reduced by 40% compared to electrochromic windows, and for cooling during the summer by 49% compared to roller shades.

Raphael and colleagues envisage a scaled up version of this device, exploiting cheap and environmentally friendly fluids, and integrating machine learning techniques for system control. Such structures could be applied as future building facades for selective tuning of light absorption and reflection to sustainably optimize the indoor climate.

The original article can be found at: Kay, R., Jakubiec, A., Katryz, C., Hatton, B. D.; Multilayered optofluidics for sustainable buildings. *Proc. Natl Acad. Sci. USA*, **120**, e2210351120; 10.1073/pnas.2210351120; 2023.

